# Comparison of analgesic effects between betamethasone and dexamethasone in total knee arthroplasty: a prospective randomized controlled trial

**DOI:** 10.3389/fmed.2025.1575417

**Published:** 2025-07-09

**Authors:** Kai Qin, Xiangxiang Sun, Qunli Dou, Bowei Li, Guoyang Bai, Xiaobo Sun, Jianbing Ma, Chao Xu, Yuanchi Huang

**Affiliations:** ^1^Department of Knee Joint Surgery, Honghui Hospital, Xi’an Jiaotong University, Xi’an, Shaanxi, China; ^2^The First Clinical Medical College, Shaanxi University of Chinese Medicine, Xianyang, China

**Keywords:** total knee arthroplasty, cocktail therapy, pain, betamethasone, dexamethasone

## Abstract

**Background:**

Corticosteroids such as dexamethasone and betamethasone are widely used for local infiltration analgesia in total knee arthroplasty (TKA). However, the analgesic efficacy of these two glucocorticoids in TKA cocktail therapy remains unknown. Therefore, this study aims to compare the analgesic efficacy and safety of betamethasone and dexamethasone in TKA through a prospective randomized controlled trial (RCT).

**Methods:**

A total of 120 patients were randomly assigned to three groups: Control (Con) group, Dexamethasone (Dex) group and Betamethasone (Beta) group. The primary outcome was the postoperative visual analog scale (VAS); the secondary outcomes were the knee range of motion (ROM).

**Results:**

Compared with the Con group, the VAS scores of the Dex group during walking were decreased significantly at 12 and 24 h postoperatively, while the Beta group showed a decrease at 12, 24, 48 h and 2 weeks postoperatively. In terms of dynamic VAS scores at 12, 24, 48 h and 2 weeks postoperatively the Beta group was superior to the Dex group. At 48 h and 2 weeks postoperatively, the ROM in the Beta group was better than Dex group. There were no significant differences among the three groups in terms of inflammatory markers and incidence of postoperative adverse event.

**Conclusion:**

Our prospective RCT demonstrates that betamethasone exhibits better analgesic effects than dexamethasone in the cocktail therapy of TKA and does not incur additional complications, providing a medication basis for the local application of glucocorticoids in TKA.

**Clinical trial registration:**

https://www.chictr.org.cn, identifier ChiCTR2300072533.

## 1 Introduction

Knee osteoarthritis (KOA) is one of the most common degenerative diseases in the elderly, mainly characterized by degeneration and loss of articular cartilage, as well as other irreversible structural changes such as osteophyte formation at the joint edge and subchondral bone (1, 2). Total knee arthroplasty (TKA) is one of the important surgical methods for end-stage KOA in elderly patients, which is used in cases who have poor responses to non-surgical and other conservative treatments (3, 4). Although TKA can significantly improve knee joint function, alleviate patient pain, and enhance their quality of life, the surgical trauma is significant and varying degrees of postoperative pain affect the recovery of the affected limb (5, 6). The persistent pain after TKA not only hinders the patient’s functional recovery and prolongs hospitalization length, but also increases postoperative medical expenses and incurs potential postoperative complications. About 20%–30% of patients express dissatisfaction with the outcome of TKA, mainly due to persistent pain and incomplete functional recovery (7, 8). The essence of optimizing analgesia in the Enhanced Recovery After Surgery (ERAS) concept is multimodal analgesia (9). The use of multimodal analgesia in TKA has achieved favorable clinical results, not only reducing postoperative pain, but also promoting early recovery and improving surgical satisfaction.

The cocktail of local infiltration analgesia around joints has been widely used in clinical practice as a component of multimodal analgesia. Numerous studies (5, 10, 11) have shown that the cocktail therapy produces favorable clinical efficacy in TKA, with long analgesic duration and few adverse reactions. Cocktails are prepared by mixing various drugs, often with local anesthetics as the main ingredients, among with glucocorticoids, adrenaline, and opioids. Corticosteroids, as a key ingredient in cocktails, have potent anti-inflammatory and analgesic effects, which can reduce surgical stress response, alleviate swelling and pain, and increase the duration of local analgesia (12, 13). In addition, the clinical application safety of corticosteroids has been validated in previous studies (14, 15).

The commonly used glucocorticoids in orthopedic cocktail formulations are dexamethasone and betamethasone, both of which have shown promising clinical efficacy in periarticular infiltration analgesia during TKA (16, 17). In terms of brachial plexus block, the addition of betamethasone can relieve pain after arthroscopic rotator cuff repair surgery (18). The application of betamethasone local infiltration in spinal surgery can significantly alleviate postoperative pain after laminoplasty and laminectomy (19). Betamethasone also exhibits better analgesic effects than dexamethasone in epidural steroid injection (ESI) (20). However, the analgesic efficacy of dexamethasone and betamethasone in the cocktail therapy of TKA is still unknown. Therefore, this study aims to evaluate the analgesic effect and safety of two glucocorticoids in TKA through clinical prospective randomized controlled trial (RCT), providing a theoretical basis for clinical medication.

## 2 Materials and methods

### 2.1 Study design

This study was designed as a prospective randomized controlled trial (RCT) comparing the efficacy of dexamethasone and betamethasone cocktail therapy on clinical outcomes of patients receiving primary TKA. This study was approved by the Ethics Committee of Xi’an Honghui Hospital (approval number: 202305007) and registered with the China Clinical Trial Registry (registration number ChiCTR2300072533). All patients provided written informed consent.

### 2.2 Participant recruitment

A total of 120 patients who underwent unilateral TKA in the knee joint ward of the Joint Surgery Department of Xi’an Honghui Hospital from June 2023 to April 2024 were recruited as the study subjects. According to the random number table, patients were randomly assigned to three groups: control group (Con), Dex group, and Beta group, with 40 cases in each group.

Inclusion criteria: (1) patients complying with the diagnostic criteria for KOA established by the American College of Rheumatology (21); (2) having an American Society of Anesthesiologists (ASA) functional status of I or II (22); (3) diagnosed with KOA before surgery, mainly unilateral symptoms, with no response to conservative treatment; (4) planning to undergo unilateral TKA; (5) aged 50–80 years; (6) not allergic to the drugs in this study; (7) willing to provide written informed consent and comply with the study procedures.

Exclusion criteria: (1) patients undergoing bilateral TKA, unicompartmental knee arthroplasty or revision surgery; (2) having severe lesions in both knee joints; (3) with cardiovascular diseases, diabetes, poor perioperative blood glucose control (fasting blood glucose > 8 mmol/L), severe renal insufficiency, severe liver insufficiency, history of gastric ulcer, and coagulation disorders; (4) history of stroke or neurological or psychiatric disorders (severe mental illness, psychological problems, and inability to communicate); (5) previous drug addiction, history of drug use, history of dependence on narcotic drugs, drug abuse, and current use of steroid drugs for treatment; (6) allergic to the drugs used in this study and experience itching and ulceration of the skin at the treatment site; (7) severe ligament instability; (8) central and peripheral nervous system issues that prevent the completion of postoperative rehabilitation plans.

### 2.3 Intervention measures

Participants were randomly assigned to the control (Con) group, dexamethasone (Dex) group, or betamethasone (Beta) group. Con group: 100 mg of 1% ropivacaine (Ruiyang Pharmaceutical Co., Ltd.; Shandong, China; 10 mL: 100 mg), diluted to 40 mL with 0.9% sodium chloride injection. Dex group: 100 mg of 1% ropivacaine + 10 mg (2 mL) dexamethasone sodium phosphate injection (Rongsheng Pharmaceutical Co., Ltd., Sino Pharm; Jiaozuo, Henan, China, 1 mL: 5 mg), diluted to 40 mL with 0.9% sodium chloride injection. Beta group: 100 mg of 1% ropivacaine + 10.52 mg (2 mL) betamethasone sodium phosphate injection (Shandong Shenglu Pharmaceutical Co., Ltd.; Shandong, China; 1 mL: 5.26 mg), diluted to 40 mL with 0.9% sodium chloride injection. All three groups of patients were injected with 40 mL of cocktail into the joint capsule of the lateral collateral ligament, quadriceps femoris, soft tissue around the patella, infrapatellar fat pad, and posterior joint capsule, as well as the soft tissue around the incision, before the insertion of the prosthesis.

### 2.4 Perioperative management

All patients followed the same perioperative protocol. After admission, the patients and their family members were informed of the ERAS plan. Health promotion and education were conducted before the surgery. Preoperative evaluation was conducted according to the patient’s individual condition, and routine preoperative examinations and preparations were completed. The patients were informed of the surgical plan and postoperative rehabilitation exercise methods. Psychological counseling was provided to improve patient confidence in the surgery and alleviate anxiety. All three groups of patients were fasted for 10 h before surgery. Symptomatic treatment was given for the underlying diseases, and blood pressure and blood sugar levels were controlled. No catheterization was required before surgery. After awakening from anesthesia, the patient was sent to the ward. The patient was allowed to drink water 2 h after surgery in the absence of obvious nausea or vomiting, and the semi-liquid diet was gradually transited to the regular diet 4 h after surgery. Intermittent ice compress (30 min each time, once every 3 h) was performed around the knee joint within 24 h after surgery to alleviate pain, limb swelling, and blood loss. All patients were required to move their ankle joints immediately after surgery. After 24 h, the patients were guided to perform knee joint range of motion (ROM) exercise, quadriceps muscle strength training, and machine passive assisted exercise, and received air pressure wave therapy to prevent thrombosis. Meanwhile, the patients were also instructed to get out of bed and walk. After surgery, a unified treatment and analgesia plan was applied. An analgesic pump was used for patient-controlled analgesia (Dezocine, 0.5 mg/mL, 100 mL) (Dezocine Injection; Yangtze River Pharmaceutical Group Co., Ltd.; Jiangsu, China; 1 mL: 5 mg). If pain persisted after removal, the painkiller Imrecoxib tablets were administered orally (0.1 g/time, twice a day) (Chengdu Shengdi Medical Co., Ltd.; Sichuan, China; 0.1 g/tablet). Low molecular weight heparin calcium injection (0.4 mL/time, once a day) was subcutaneously injected after surgery (Hebei Changshan Biochemical Pharmaceutical Co., Ltd.; Hebei, China; 0.4 mL: 4100AXaIU) to prevent embolism, which was maintained until discharge 3 days after surgery. After discharge, the patients were instructed to take rivaroxaban tablets (2.5 mg/time, once a day) (Qilu Pharmaceutical Co., Ltd.; Shandong, China; 10 mg/tablet) orally for 10–14 days. The patients were required to continue quadriceps strength training and knee joint ROM exercise after discharge and to undergo regular follow-up examinations until knee functional recovery.

### 2.5 Surgical procedures

All surgeries were performed in collaboration with a team of experienced doctors. After delivered to the operating room, the patient was placed in a supine position and given routine oxygen therapy. Vital signs such as blood pressure, electrocardiogram, and blood oxygen saturation were monitored, and general anesthesia with laryngeal mask and femoral nerve block were administered. After satisfactory anesthesia induction, routine disinfection and surgical drapes were applied. An inflatable tourniquet was used with a pressure of 38 kPa. Through a conventional patellar medial approach, the joint capsule was incised, followed by precise osteotomy and installation of trial force line. Then, the flexion extension gap was balanced and the hypertrophic osteophyte was cleaned. After the trial, the surgical area was rinsed with type III iodine and sterile normal saline pulse pressure gun, and 40 mL of prepared cocktail was injected around the joint. The patients were fitted with the same prosthesis (Beijing AK Medical Co., Ltd., Beijing, China), straightened the knee joint, and waited for the bone cement to solidify. Afterward, the tourniquet was loosened and the surgical area was washed again with type III iodine and sterile normal saline pulse pressure gun, and then, the wound was sutured layer by layer. No wound drainage tube was placed, and an elastic bandage was applied with appropriate pressure for bandaging.

### 2.6 Primary outcomes

Analgesic effect: three groups of patients were compared for visual analog scale (VAS) scores at 6, 12, 24, 48 h and 2 weeks after TKA surgery at rest and exercise (passive functional exercise) states (23). VAS scores ranged from 0 to 10 points, with 1 to 3 points indicating no pain or mild pain, 4 to 7 points indicating pain that affects rest but can be tolerated, and 8 to 10 points indicating severe pain. The higher the score, the more severe the pain.

### 2.7 Secondary outcomes

Prior to surgery, an angle measuring instrument was used to measure the knee ROM at 24, 48 h, and 2 weeks postoperatively. Preoperative C-reactive protein (CRP), erythrocyte sedimentation rate (ESR), and interleukin-6 (IL-6) levels in the blood were measured at 24 and 48 h postoperatively, reflecting the inflammatory response. In this study, inflammatory marker data were obtained by a professional team from the Clinical Laboratory Department of Xi’an Honghui Hospital following standardized clinical testing protocols. All measurements were conducted in compliance with institutional ethical review and quality control standards. Peripheral venous blood samples were collected from each patient for the measurement of CRP, IL-6 levels, and ESR. Blood samples were centrifuged at 3,000 rpm for 10 min at room temperature, and the resulting serum was subjected to further analysis. CRP levels and ESR were determined using an automated biochemical analyzer (Mindray, BS-280) employing enhanced immunoturbidimetric assay and Westergren method, respectively. Serum interleukin-6 (IL-6) levels were measured via enzyme-linked immunosorbent assay (ELISA) using the same analyzer (Mindray, BS-280). All ELISA kits were procured from Quanyuan Biotechnology Center (Shanghai, China), and assays were performed strictly in accordance with the manufacturer’s instructions.

The time required for straight leg raise (SLR) after TKA was recorded in three groups of patients.

The adverse reactions such as postoperative nausea and vomiting, wound aseptic fat liquefaction, surgical site infection, hematoma, deep vein thrombosis, pulmonary embolism, and readmission were also recorded.

### 2.8 Sample size and statistical analysis

The postoperative VAS score was chosen as the primary outcome. Sample size estimation was based on a preliminary study conducted on 21 patients (seven patients per group) who underwent unilateral TKA for the first time. Sample size estimation was conducted using G Power software version 3.1.9. For the 1:1:1 parallel control trial, under the assumption of the type I error rate = 5%, the sample size of 38 patients in each group was required to achieve 90% confidence. Fixed effect model and one-way analysis of variance (ANOVA) design were adopted. The estimated attrition rate was 10%, so we planned to recruit 42 patients in each group, with a total of 126 patients.

The data were processed using SPSS29.0 statistical software. The measurement data were subjected to exploratory analysis and tested for normal distribution, with a test standard of α = 0.05, and all tests were two-sided. The measurement data were described as mean ± s.d. (x ± s), while the count data were described as example (%). The demographic data of three groups of patients, including age, body mass index (BMI), preoperative dynamic VAS score, and preoperative joint ROM, were analyzed using one-way ANOVA or Kruskal-Wallis test. The gender data were analyzed using χ^2^ test. The continuous data in clinical outcomes, including VAS, joint ROM, and blood related indicators, were analyzed using Kruskal-Wallis test. The required time for the SLR test and the number of postoperative adverse reactions were determined using the χ^2^ test or Fisher’s exact probability method. A value of *P* < 0.05 indicated a significant difference.

## 3 Results

### 3.1 Baseline characteristics

**FIGURE 1:**
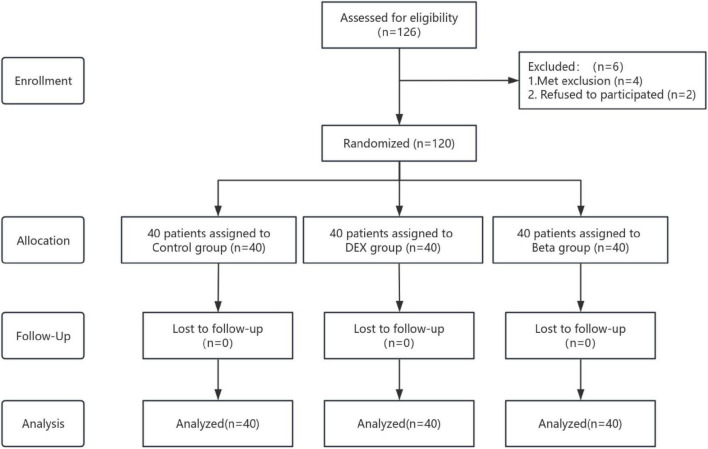
Study flow chart.

A total of 126 patients who planned to undergo primary unilateral TKA were enrolled in this trial. After further screening, four patients were excluded according to the exclusion criteria and two patients refused to participate in this trial. Finally, 120 patients were randomly assigned to three groups (Figure 1). There were no significant differences in demographic characteristics, preoperative dynamic VAS scores, and joint ROM among the three groups, indicating comparability (Table 1).

**TABLE 1:** Characteristics of the patients at baseline.

Variable	Con group (*n* = 40)	Dex group (*n* = 40)	Beta group (*n* = 40)	*P*-value
Gender				
Male	15	15	16	0.965
Female	25	25	24	
Age, years	66.25 ± 5.87	68.00 ± 6.16	66.73 ± 6.33	0.420
BMI, kg/m^2^	25.55 ± 3.31	25.62 ± 2.88	25.06 ± 3.25	0.691
VAS at movement of Pre-OP	5.65 ± 0.83	5.60 ± 0.81	5.76 ± 0.90	0.922
ROM of Pre-OP	106.2 ± 13.64	102.4 ± 11.09	103.16 ± 11.21	0.325

BMI, body mass index; VAS, visual analog score; ROM, range of motion; Pre-OP, preoperative; *P*-value, indicates a significant difference among the groups.

### 3.2 Analgesic effect (VAS)

**FIGURE 2:**
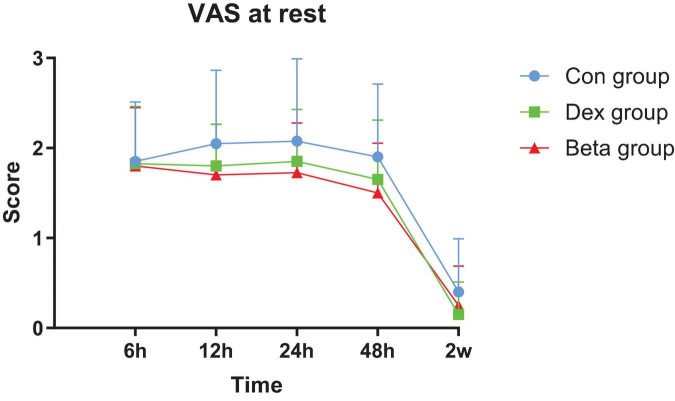
Postoperative visual analog scale (VAS) at rest.

**FIGURE 3:**
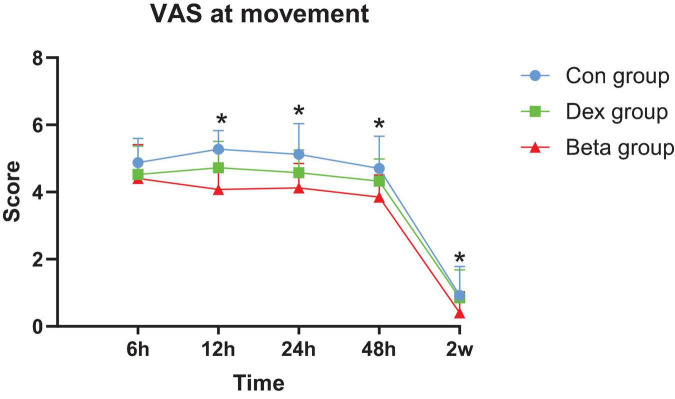
Postoperative visual analog scale (VAS) at movement. *, dex group vs. beta group; *P* < 0.05 is significant.

At rest state, there was no difference in VAS scores among the three groups at all postoperative time points. During walking, compared with the Con group, the Dex group showed a decrease in VAS scores at 12 and 24 h after surgery (4.73 ± 0.78, 4.58 ± 0.68), while the Beta group showed a decrease in VAS scores at 12, 24,48 h and 2 weeks after surgery (4.08 ± 1.14, 4.13 ± 0.72, 3.85 ± 0.66, 0.40 ± 0.63), with statistically significant differences (*P*_1_ = 0.008, *P*_1_ = 0.036, *P*_2_ = 0.000, *P*_2_ = 0.000, *P*_2_ = 0.000, *P*_2_ = 0.012). In addition, the dynamic VAS scores at 12, 24, 48 h and 2 weeks after surgery were better in the Beta group than those in the Dex group (4.08 ± 1.14 vs. 4.73 ± 0.78, 4.13 ± 0.72 vs. 4.58 ± 0.68, 3.85 ± 0.66 vs. 4.33 ± 0.66,0.40 ± 0.63 vs. 0.85 ± 0.83), with statistically significant differences (*P*_3_ = 0.044, *P*_3_ = 0.040, *P*_3_ = 0.016, *P*_3_ = 0.037) (Table 2, Figures 2, 3).

**TABLE 2:** Postoperative pain evaluation.

Duration	Con group (*n* = 40)	Dex group (*n* = 40)	Beta group (*n* = 40)	*P*-value	*P*_1_	*P*_2_	*P*_3_
**VAS at rest**							
Post-op 6 h	1.85 ± 0.66	1.83 ± 0.64	1.80 ± 0.65	0.909	1.000	1.000	1.000
Post-op 12 h	2.05 ± 0.82	1.80 ± 0.46	1.70 ± 0.57	0.116	0.700	0.117	1.000
Post-op 24 h	2.08 ± 0.92	1.85 ± 0.58	1.73 ± 0.55	0.218	1.000	0.243	1.000
Post-op 48 h	1.90 ± 0.81	1.65 ± 0.66	1.50 ± 0.56	0.074	0.524	0.070	1.000
Post-op 2 weeks	0.40 ± 0.59	0.15 ± 0.36	0.25 ± 0.43	0.100	0.096	0.767	0.939
**VAS at movement**							
Post-op 6 h	4.88 ± 0.72	4.53 ± 0.85	4.40 ± 1.01	0.057	0.210	0.071	1.000
Post-op 12 h	5.28 ± 0.55	4.73 ± 0.78	4.08 ± 1.14	<0.001*	0.008*	0.000*	0.044*
Post-op 24 h	5.13 ± 0.91	4.58 ± 0.68	4.13 ± 0.72	<0.001*	0.036*	0.000*	0.040*
Post-op 48 h	4.70 ± 0.97	4.33 ± 0.66	3.85 ± 0.66	<0.001*	0.219	0.000*	0.016*
Post-op 2 weeks	0.93 ± 0.86	0.85 ± 0.83	0.40 ± 0.63	0.008	1.000	0.012*	0.037*

VAS, visual analog score; Post-op, postoperative; *P*-value, indicates a significant difference among the groups; *P*_1_, con group vs. dex group; *P*_2_, con group vs. beta group; *P*_3_, dex group vs. beta group; *, *P* < 0.05 is significant.

### 3.3 Knee range of motion (ROM)

**FIGURE 4:**
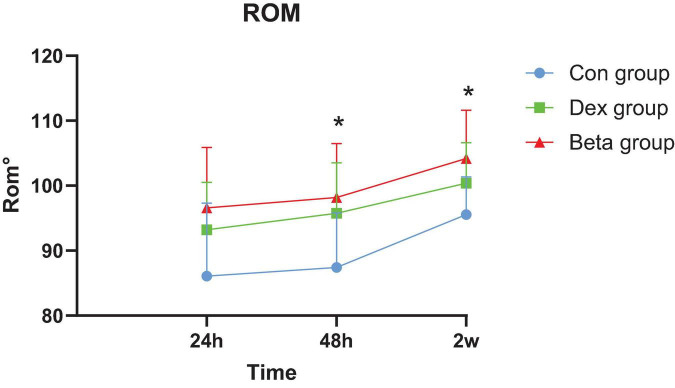
Postoperative knee range of motion. *, dex group vs. beta group; *P* < 0.05 is significant.

Compared with the Con group, the Dex group (93.21 ± 7.32, 95.76 ± 7.75, 100.40 ± 6.23) and Beta group (96.59 ± 9.28, 100.14 ± 6.63, 104.19 ± 7.44) showed an increase in knee ROM at 24, 48 h and 2 weeks after surgery, with statistical differences (*P*_1_ = 0.039, *P*_1_ = 0.001, *P*_1_ = 0.004, *P*_2_ = 0.000, *P*_2_ = 0.000, *P*_2_ < 0.001). At 48 h and 2 weeks after surgery, the knee ROM in the Beta group was better than that in the Dex group (100.14 ± 6.63 vs. 95.76 ± 7.75, 104.19 ± 7.44 vs. 100.40 ± 6.23), with a statistically significant difference (*P*_3_ = 0.033, *P*_3_ = 0.032) (Table 3, Figure 4).

**TABLE 3:** Postoperative knee range of motion (ROM) (°).

Duration	Con group (*n* = 40)	Dex group (*n* = 40)	Beta group (*n* = 40)	*P*-value	*P*_1_	*P*_2_	*P*_3_
Post-op 24 h	86.09 ± 11.21	93.21 ± 7.32	96.59 ± 9.28	<0.001*	0.039*	0.000*	0.390
Post-op 48 h	87.43 ± 8.41	95.76 ± 7.75	100.14 ± 6.63	<0.001*	0.001*	0.000*	0.033*
Post-op 2 weeks	95.57 ± 5.79	100.40 ± 6.23	104.19 ± 7.44	<0.001*	0.004*	<0.001*	0.032*

Post-op, postoperative; *P*-value, indicates a significant difference among the groups; P_1_, con group vs. dex group; *P*_2_, con group vs. beta group; *P*_3_, dex group vs. beta group; *, *P* < 0.05 is significant.

### 3.4 Straight leg raise (SLR)

The number of patients who completed the SLR test in the Dex and Beta groups was higher than that in the Con group at 12, 24, 48 h after surgery, and the time for the first SLR was shorter, with statistical significance differences (*P* = 0.002, *P* < 0.001) (Table 4).

**TABLE 4:** Postoperative the number of straight leg raise (*n*, %).

Duration	Con group (*n* = 40)	Dex group (*n* = 40)	Beta group (*n* = 40)	*P*-value	*P*_1_	*P*_2_	*P*_3_
Post-op 12 h	9 (22.5)	20 (50.0)	24 (60.0)	0.002*	0.011**	<0.001**	0.369
Post-op 24 h	22 (55.0)	32 (80.0)	36 (90.0)	<0.001*	0.017**	<0.001**	0.210
Post-op 48 h	27 (67.5)	35 (87.5)	38 (95.0)	0.003*	0.032*	0.002*	0.235
Post-op 2 weeks	40 (100.0)	40 (100.0)	40 (100.0)	1.000	1.000	1.000	1.000

Post-op, postoperative; *P*-value, indicates a significant difference among the groups; *, *P* < 0.05 is significant; **, *P* ≤ 0.01 is significant.

### 3.5 Inflammatory indicators

There were no statistically significant differences in the levels of CRP, ESR, and IL-6 among the three groups before surgery, at 24 and 48 h after surgery (Table 5).

**TABLE 5:** Preoperative and postoperative inflammatory indicators.

Duration	Con group (*n* = 40)	Dex group (*n* = 40)	Beta group (*n* = 40)	*P*-value	*P*_1_	*P*_2_	*P*_3_
**CRP (mg/L)**							
Pre-OP	1.80 @ 1.77	2.52 @ 2.70	2.54 @ 2.74	0.518	0.957	0.967	1.000
Post-op 24 h	25.42 @ 20.87	18.86 @ 12.49	16.92 @ 17.80	0.062	1.000	0.061	0.389
Post-op 48 h	72.98 @ 47.15	55.69 @ 46.14	50.29 @ 36.35	0.053	0.131	0.089	1.000
**ESR (mm/H)**							
Pre-OP	11.85 @ 8.70	11.70 @ 7.93	9.90 @ 6.61	0.600	1.000	1.000	1.000
Post-op 24 h	18.88 @ 13.10	18.60 @ 11.80	14.08 @ 9.25	0.161	1.000	0.392	0.231
Post-op 48 h	30.90 @ 11.94	29.00 @ 16.81	24.95 @ 11.24	0.111	0.638	0.112	1.000
**IL-6 (pg/mL)**							
Pre-OP	1.72 @ 0.67	2.05 @ 2.59	1.88 @ 1.09	0.978	1.000	1.000	1.000
Post-op 24 h	81.48 @ 64.49	61.05 @ 56.66	55.89 @ 43.93	0.055	0.082	0.160	1.000
Post-op 48 h	52.20 @ 43.78	32.50 @ 28.93	34.60 @ 25.97	0.065	0.075	0.272	1.000

CRP, C-reactive protein; IL-6 level, interleukin-6; ESR, erythrocyte sedimentation rate; Pre-OP, preoperative; Post-op, postoperative; *P*-value, indicates a significant difference among the groups; *P*_1_, con group vs. dex group; *P*_2_, con group vs. beta group; *P*_3_, dex group vs. beta group.

### 3.6 Postoperative adverse reactions

There was no statistically significant difference in the incidence of postoperative adverse reactions among the three groups (Table 6).

**TABLE 6:** Postoperative complications (*n*, %).

Variable	Con group (*n* = 40)	Dex group (*n* = 40)	Beta group (*n* = 40)	*P*-value
Nausea and vomiting	11 (27.5)	10 (25.0)	7 (17.5)	0.546
Aseptic fat liquefaction of wound	2 (5.0)	1 (2.5)	0	0.772
Hematoma	0	0	0	N/A
Surgical area infection	0	0	0	N/A
Deep vein thrombosis	3 (7.5)	1 (2.5)	1 (2.5)	0.617
Pulmonary embolism	0	0	0	N/A
Readmission	0	0	0	N/A

*P*-value, indicates a significant difference among the groups; N/A, not applicable.

## 4 Discussion

The surgical processes of TKA involve bone resection and soft tissue release, which can insult persistent postoperative pain (24, 25). The mainstream postoperative pain control methods for TKA include epidural analgesia, patient-controlled analgesia (PCA), peripheral nerve block, femoral nerve block (FNB), adductor block (ACB), and local invasive analgesia (LIA) (26). However, postoperative pain remains a major clinical challenge after TKA. Postoperative pain can significantly compromise early recovery, prolong hospitalization length, intensify economic burden, increase opioid consumption, and reduce surgical satisfaction, which does not conform to the concept of ERAS (26, 27).

Enhanced Recovery After Surgery represents an evidence-based, multimodal perioperative approach designed to minimize surgical stress, enhance physiological function, and reduce postoperative morbidity and mortality, thereby improving surgical safety, patient satisfaction, and recovery rates. Pain management serves as a cornerstone of this paradigm (28–30). In this study, betamethasone and dexamethasone were integral components of the ERAS protocol (31). The comparative evaluation of these glucocorticoids was motivated by the ERAS principle of optimizing analgesic strategies. First, local glucocorticoid infiltration complements nerve blocks and non-steroidal anti-inflammatory drugs (NSAIDs) by targeting inflammatory mediators (16), thereby reducing opioid requirements and associated adverse effects (e.g., nausea, ileus) (32). This synergy facilitates early oral intake and mobilization. Second, betamethasone’s prolonged analgesic duration (16) aligns with ERAS objectives, particularly the emphasis on early functional rehabilitation. Moreover, its localized administration circumvents systemic metabolic disturbances (e.g., hyperglycemia) (31, 32), ensuring unimpeded implementation of other ERAS measures (e.g., same-day ambulation). Finally, elucidating the potency differential between these agents (33) enables dose individualization based on patient characteristics (e.g., diabetes, obesity), further refining ERAS practices.

Local infiltration injection with cocktail analgesics around the joints is effective in reducing pain and promoting early functional improvement after TKA (34). Corticosteroids, as an important component of cocktails, exert strong anti-inflammatory and analgesic effects by inhibiting pro-inflammatory cytokines and inducing anti-inflammatory cytokines, reducing prostaglandin synthesis, and thereby diminishing the excitability of local pain receptors (35–37). Dexamethasone and betamethasone are the two most commonly used high-efficiency and long-acting glucocorticoids in multimodal cocktails, with the characteristics of easy absorption and fast onset. In this study, we demonstrated that both types of glucocorticoids exhibited favorable analgesic effects and safety after TKA. In addition, compared to dexamethasone, betamethasone further reduced patient pain and achieved earlier SLR 12–48 h after surgery in dynamic mode.

Steroids can be divided into particles and non-particles. Dexamethasone only exists in non-particulate form in saline solution (38), and its effect is most pronounced within the first 24–48 h (39), but it fails to maintain an effective concentration for a long period of time. Betamethasone, as a stereoisomer of dexamethasone, has a biological half-life of 36–55 h. Betamethasone due to differences in molecular structure and pharmaceutical formulation, partially exists in the form of particles in ropivacaine as a local reserve, thus possessing more persistent anti-inflammatory and analgesic properties (18), betamethasone exhibits superior analgesic efficacy. Betamethasone is the C16β-methyl epimer of dexamethasone, demonstrating slightly higher glucocorticoid receptor (GR) binding affinity (potency ratio ∼1.3:1), which may enhance its local anti-inflammatory effects (40). The dual-salt formulation of betamethasone (sodium phosphate + acetate) combines rapid onset (sodium phosphate) with sustained release (acetate), whereas dexamethasone’s single-salt formulation (sodium phosphate) has a shorter duration of action, potentially limiting its postoperative analgesic persistence (41). Glucocorticoid dosing must account for potency conversion: 1 mg betamethasone ≈ 6.5–7 mg dexamethasone (based on anti-inflammatory equivalence). Failure to adjust doses proportionally in comparative studies may underestimate betamethasone’s potency advantage (33). In this trial, betamethasone (10.52 mg) and dexamethasone (10 mg) were not dose-adjusted for equivalence. According to conversion standards, 10 mg dexamethasone corresponds to 1.4–1.5 mg betamethasone; thus, 10.52 mg betamethasone likely exerted significantly stronger anti-inflammatory and analgesic effects. Betamethasone’s plasma half-life (∼6.3 days) exceeds that of dexamethasone (∼36–72 h). Its acetate component forms a “drug depot” at the injection site due to low solubility, enabling prolonged local anti-inflammatory activity via slow release (42, 43). Additionally, betamethasone’s particulate nature (versus dexamethasone’s non-particulate form) may enhance penetration and retention in synovial and periarticular tissues, more effectively suppressing pro-nociceptive mediators (e.g., PGE2, IL-6). In our study, we found that compared with the Con group, the VAS scores of the Dex group in dynamic mode only showed statistical differences at 12 and 24 h after surgery, while the VAS scores of the Beta group were significantly decreased at all time points except 6 h, demonstrating better long-term efficacy of betamethasone. Luo et al. (16) compared 50 patients who received ropivacaine alone and 50 patients who received knee joint injections of ropivacaine, betamethasone, and morphine. The results showed that the VAS scores of the group receiving betamethasone injection were lower than those of the control group. Liu et al. (44) found that local infiltration injection of a cocktail containing betamethasone in unicompartmental knee arthroplasty (UKA) resulted in a significant decrease in VAS scores between 18 and 48 h postoperatively compared to the injection without betamethasone cocktail, demonstrating the efficacy of low-dose betamethasone in relieving acute postoperative pain. These findings are similar to our results suggesting that betamethasone can significantly alleviate pain after TKA surgery. In addition, the VAS scores of the Beta group at 12, 24, 48 h and 2 weeks after surgery were lower than those of the Dex group, indicating that betamethasone is superior to dexamethasone in pain relief in TKA cocktail therapy.

Range of motion is a pivotal indicator for evaluating the early functional recovery of patients after TKA, and adequate functional exercise in the early postoperative period can also preserve middle and long-term knee ROM to a greater extent after surgery (36). However, lower limb swelling caused by surgical trauma and local inflammation can affect the postoperative ROM of the knee joint. Corticosteroids can inhibit capillary dilation, alleviate tissue edema and exudation in the early stages of inflammation (36). In this study, both the Beta group and Dex group showed better ROM at 24, 48 h and 2 weeks after surgery compared to the Con group. Luo et al. (16). demonstrated that adding morphine and betamethasone to a local infiltration analgesia (LIA) mixture enhanced early knee functional recovery. Similarly, Zheng et al. (36) found that patients who received local application of betamethasone had significantly improved maximum extension angle, maximum flexion angle, and passive ROM on the 3rd day after TKA compared to the controls. Wang et al. (45) also revealed that the postoperative functional recovery of the glucocorticoid group was significantly better than that of the control group. Moreover, the ROM of the Beta group was better than that of the Dex group at 48 h and 2 weeks after surgery, in consistency with the VAS score results, suggesting that better joint ROM may be associated with better pain relief.

Straight leg raise is a common functional exercise after TKA, reflecting the strength of the quadriceps femoris muscle (46). The standard for postoperative SLR test depends on the patient’s muscle strength and pain. On the one hand, lower limb swelling after TKA is believed to reduce voluntary muscle activation through a process called arthrogenous muscle inhibition (AMI) and thus lowers muscle strength (4). On the other hand, pain can affect lower limb activities, including SLR. Hence, better muscle strength is associated with stronger analgesic effect and earlier completion time of SLR. In this study, the number of patients in the Beta group and Dex group who performed SLR at 12, 24, 48 h was significantly higher than that in the Con group, indicating that the use of glucocorticoids can shorten the time for postoperative SLR and this advantage is related to pain relief. Ikeuchi et al. (47), Luo et al. (16) demonstrated a significant increase in the number of individuals who completed SLR earlier in the glucocorticoid group. However, Bagheri Fard et al. (48) pointed out that in TKA, there was no significant difference in knee ROM and SLR between patients receiving corticosteroid injection around joints and controls. The inconsistent conclusions may be related to differences in cocktail recipes.

C-reactive protein, ESR, and IL-6, as inflammatory markers, exhibit similar dynamic changes during inflammation, and their levels are positively correlated with acute pain after initial TKA (49). Steroids reduce the production of cyclooxygenase and lipoxygenase by inhibiting phospholipase, thereby reducing the mediators of systemic inflammation and acute stress response (5). The analgesic mechanism of local application of glucocorticoids is the direct inhibition of C-fiber signal transduction, rather than the reduction of inflammation (50, 51). Therefore, local infiltration containing glucocorticoids can alleviate pain but does not reduce systemic inflammatory markers. This also explains why there was no difference in postoperative inflammation indicators among the three groups of patients in our study.

In this study, there were 28 cases of nausea and vomiting, three cases of fat liquefaction, and four cases of deep vein thrombosis. There was no significant difference in the incidence of adverse reactions among the three groups, and no adverse events such as hematoma, surgical site infection, or pulmonary embolism were found. Multiple studies have shown (16, 47) that there is no significant increase in adverse reactions in patients receiving local injection of corticosteroid cocktails, which is similar to our results. In addition, the incidence of side effects of glucocorticoids is related to dose and time. In this study, glucocorticoids were applied locally at low doses, and no serious adverse events occurred.

There are several limitations to this study. Firstly, this study did not grade the severity of KOA in the participating patients. Different degrees of osteophyte clearance and soft tissue release during surgery can cause varying degrees of surgical trauma, which may affect the observation results of pain. fly, this study focused on perioperative analgesia, while the follow-up time was relatively short. Hence, the long-term analgesic action and side effects of betamethasone are still unknown. Thirdly, all cases in this study were from an orthopedic hospital in Asia with high surgical capacity. The conclusions may not be widely applicable to medical institutions in different regions of the world.

## 5 Conclusion

In conclusion, our prospective randomized controlled trial demonstrates that the analgesic effect of betamethasone is superior to dexamethasone in the cocktail therapy of TKA and does not eventuate additional complications, providing a medication basis for the local application of glucocorticoids after TKA.

## Data Availability

The raw data supporting the conclusions of this article will be made available by the authors, without undue reservation.
